# Sett priming with salicylic acid improves salinity tolerance of sugarcane (*Saccharum officinarum* L.) during early stages of crop development

**DOI:** 10.1016/j.heliyon.2023.e16030

**Published:** 2023-05-06

**Authors:** Tasfiqure Amin Apon, Sheikh Faruk Ahmed, Zannatul Ferdaous Bony, Md. Rizvi Chowdhury, Jannatul Ferdoushi Asha, Arindam Biswas

**Affiliations:** a Pathology Division, Bangladesh Sugarcrop Research Institute (BSRI), Ishurdi, 6620, Pabna, Bangladesh; b Department of Crop Botany, Bangabandhu Sheikh Mujibur Rahman Agricultural University (BSMRAU), Gazipur, 1706, Bangladesh; c Department of Agroforestry and Environment, Bangabandhu Sheikh Mujibur Rahman Agricultural University (BSMRAU), Gazipur, 1706, Bangladesh; d Department of Plant Pathology, Bangabandhu Sheikh Mujibur Rahman Agricultural University (BSMRAU), Gazipur, 1706, Bangladesh; e Department of Agricultural Chemistry, Hajee Mohammad Danesh Science and Technology University (HSTU), Dinajpur 5200, Bangladesh; f Bangladesh Agricultural Research Institute (BARI), Joydebpur, Gazipur, 1701, Bangladesh

**Keywords:** Germination, *Saccharum officinarum* L., Salt stress, Seedling vigor, Salicylic acid priming, Stress mitigation

## Abstract

Sugarcane (*Saccharum officinarum* L.), a globally cultivated carbohydrate producing crop of industrial importance is being challenged by soil salinity due to its glycophytic nature. Water stress coupled with cellular and metabolic alterations resulting from excess sodium (Na^+^) ion accumulation is irreversibly damaging during early crop developmental stages that often results in complete crop failure. This study therefore aimed to explore the potential of salicylic acid as a sett priming material to mitigate the negative effects of salt stress on sugarcane during germination and early growth stages. Five doses of salicylic acid (0 [hydropriming] [control], 0.5 mM, 1 mM, 1.5 mM and 2 mM) were tested against three levels of salinity (0.5 dS m^−1^ [control], 4 dS m^−1^, and 8 dS m^−1^) within a polyhouse environment. Results revealed 11.2%, 18.5%, 25.4%, and 38.6%, average increase in final germination, germination energy, seedling length and seedling vigor index respectively with a subsequent reduction of 21% mean germination time. Investigations during early seedling growth revealed 21.6%, 17.5%, 27.0%, 39.9%, 10.7%, 11.5%, 17.5%, 47.9%, 35.3% and 20.5% overall increase in plant height, total leaf area, shoot dry matter, root dry matter, leaf greenness, relative water content, membrane stability index, proline content, total antioxidant activity and potassium (K^+^) ion accumulation respectively with a subsequent reduction of 24.9% Na^+^ ion accumulation and 35.8% Na^+^/K^+^ ratio due to salicylic acid priming. Germination, seedling growth and recovery of physiochemical traits were highly satisfactory in primed setts than non-primed ones even under 8 dS m^−1^ salinity level. This study should provide useful information for strategizing salinity management approaches for better productivity of sugarcane.

## 1 Introduction

Soil salinity due to availability of excess exchangeable sodium (Na^+^) ions in the soil solution resulting in an electrical conductivity (EC) above 4 dS m^−1^ stands as a major abiotic stress limiting crop productivity over 100 countries of the world, covering no less than a billion hectares of cultivable lands [[1], [2], [3]]. In Asia alone, around 295 million hectares of croplands are salt affected and thereby causing crop loss worth of approximate $27.3 billion due to productivity loss as high as 97% [1,4]. Cultivable lands of coastal region are highly saline because of sea-water intrusion as the primary reason followed by anthropogenic processes [[5], [6], [7]]. Bangladesh stands highly vulnerable in this perspective as she not only holds 30% of her total cultivable land at coastal regions but also she is being threatened by climate change mediated sea-level rise [8]. Thus, salt stress is quite prominent in this country similar to most of the seashore countries of the world which could impact plants in two ways. Firstly, excess salt ions make water uptake difficult for plants via reduction of water potential of the soil solution [9] and secondly exhibits cellular toxicity via excess ion accumulation [5,[10], [11], [12]]. However, the degree of damage could vary depending on genotypic tolerance and cropping environment [13].

Sugarcane (*Saccharum officinarum* L.) is a sugar producing plant from grass family Poaceae, cultivated globally as an important industrial crop [14]. In Bangladesh, sugarcane ranks as the third most important cash crop that covers about 1.52% of the countries cultivable land area [15]. However, being a typical glycophytic plant it is moderately sensitive to salt stress especially during the early growth stages [16,17]. Moreover, sugarcane is one of the most water-requiring crops and only osmotic stress in sugarcane could result in sugar loss as high as 60% [18]. Thus, successful sugarcane cropping under salinity is comparatively inflexible as salt stress simultaneously exerts both osmotic and ionic stress [9]. Difficulty in water uptake undoubtedly would have its impact on poor germination but salt accumulation in cells would also trigger metabolic alterations, damages to membranes, organelle, important biomolecules like proteins and nucleic acids via free radicle generation ultimately resulting in weak seedling growth to seedling death depending on the degree of salinity [17,[19], [20], [21], [22]]. The hyper-ionic stress of salinity also interferes with nutrients absorption from the soil leading to plant demise [23]. Although, different sugarcane genotypes might show variability in terms of salt tolerance but almost all glycophytic plant species require managerial interventions to effectively mitigate the adverse impacts of salt stress.

Salicylic acid (SA), a phenolic compound of plant origin, was initially thought as a phytohormone because of its intricate role in plant growth promotion and tolerance against both biotic and abiotic stresses [24]. Till today, the alleviation roles of SA for a number of abiotic stresses including salinity in various plants is well established. Evidence suggests positive role of SA against salinity and many other abiotic stresses via regulation of stomatal operation, transpiration, photosynthetic pigments, and respiratory pathways [25]. For instance, Seed priming of SA arrested nodule senescence, boosted photosynthetic efficiency, up-regulated free radicle quenching machinery leading to restored biomass and yield in salt-stressed chickpea [26]. Plants exposed to abiotic stresses like drought, salinity, high and low temperature, waterlogging, and metalloid stress usually triggers metabolic and transcriptional alterations. Under the adverse situation SA was found benefiting yield-related attributes of plants via modulating physiological and biochemical responses. Balassa et al. [27] mentioned the fact that exogenous SA treatment could result in RNAi gene expression patterns in plants similar to drought tolerance responses. Because of this phenomenon they suggested that SA priming could induce RNA-dependent DNA methylation and chromatin remodeling, which may result in the faster activation of stress-responsive metabolic routes. In addition, SA application was mentioned to help growth and productivity of plants by modulating auxin and cytokinin levels in salt-stressed wheat plants that ultimately promoted cell division and elongation [28,29]. Furthermore, SA and other salicylate derivatives are also known for their endogenous signaling responses against plant abiotic stresses including salt stress where SA could regulate phytohormone (auxin, ethylene, abscisic acid, gibberellic acid, jasmonic acid, and cytokinins) biosynthesis, osmotic homeostasis, mineral nutrient uptake, free radicle scavenging, and production of compatible secondary metabolites (terpenes, phenolics, alkaloids, glucosides, glutathione, phytoalexines, glucosinolates, defensins, and thionins) [[30], [31], [32], [33], [34]]. Apart from that, recent reports also insight the critical roles of SA in eliciting physiological and molecular responses regarding salinity tolerance of plants. For instance, in pea SA induced salinity tolerance via up-regulating the expression of plasma membrane Na^+^/H^+^ antiporter *SOS1* and vacuolar Na^+^/H^+^ exchanger *NHX1* [35]. In pistachio, SA positively modulated expression of isochorismate synthase (*ICS*) gene that lead to salt stress tolerance [36]. Exogenous application of SA in forms of both foliar spray and seed priming was reported to improve salt tolerance of plants like Arabidopsis, rice, wheat, alfalfa, corn, rye and tomato [23,[37], [38], [39], [40], [41], [42], [43]]. In case of sugarcane, we found a single report by Badil et al. [44] suggesting SA application as foliar spray at early seedling stage for seedling growth enhancement under saline environment. However, due to the general practice of sugarcane cultivation via direct sett sowing; germination and seedling establishment under saline environment remains an important stage to put emphasis and to the best of our knowledge priming has been explored very little for sugarcane with no documentation on sett priming with salicylic acid.

Priming itself is an emerging strategy that has successfully been used to enhance seed germination, seedling establishment, and vigorous early vegetative growth of seedlings in several field crops like sorghum, millet and wheat under salt stress [43,45,46]. On the other hand, SA is an eco-friendly biochemical compound proven to enhance the salt tolerance of many field crops. Increasing soil salinity is a growing concern throughout the world. Given the circumstances of salt stress mediated reduction of crop productivity in the coastal belt, deployment of strategies related to salt stress mitigation is a crying need for successful crop productivity. Good germination performance and robust seedling vigor during early crop developmental stages are very important for obtaining a good yield of sugarcane. Salicylic acid is a well-known salt stress alleviator of many field crops involving physiological regulation, biochemical adjustments and gene expression modulation when applied exogenously both as foliar spray and seed priming; however, we did not come across any notable study investigating the efficacy of SA as a priming material in sugarcane salinity tolerance. Thus, a research gap was evident on which the current study emphasizes. Furthermore, due to comparatively larger size of sugarcane setts than seeds of many other popular field crops, SA priming is more likely to result in better stress alleviation and thereby aiding in sustainable crop productivity. Hence, the current study focuses on investigating the impacts of SA sett priming on salt stress tolerance of sugarcane germination, growth, physiological and biochemical attributes during the early crop developmental stages.

## 2 Materials and methods

### 2.1 Experimental materials and conditions

Single eyed setts (5 cm long) from 10 months old sugarcane (*Saccharum officinarum* L.) cultivar “BSRI Akh 42” were used in this experiment. The setts were generously provided by Bangladesh Sugarcrop Research Institute (BSRI), Ishurdi 6620, Pabna, Bangladesh. The experiment was conducted in a polyhouse of BSRI (24.1156° N, 89.0817° E), Ishurdi 6620, Pabna, Bangladesh; during March 2022. Temperature and relative humidity features of the polyhouse were somewhat between 25 and 35 °C and 65–75% respectively, throughout the experimental period. Silty clay loam soil (Ganges-river floodplain soil) consisting of around 15.6% sand, 46.1% silt, 34.3% clay, and 4.1% organic matter, available Na^+^ content of 3.68 μg g^−1^, with a pH of approximate 7.3 was collected from the research field of BSRI. Initial nutrient status of the soil has been presented in (Supplementary Table 1). The collected soil was then made friable by air drying, grinding, and sieving with 6 mm × 6 mm mesh. Plants were raised in black colored perforated plastic pots (height: 20 cm, top radius: 12 cm and bottom radius: 9 cm) filled with 10 kg of the prepared soil. Each pot received a standard fertilizer dose (Supplementary Table 2) recommended by Bangladesh Agricultural Research Council (BARC) for sugarcane cultivation using Ganges-river floodplain soil which was applied a day prior to sowing setts.

### 2.2 Experimental design and treatments

The experiment was designed following a completely randomized design (CRD) with factorial combination of three levels of salinity (0.5 dS m^−1^ [control], 4 dS m^−1^, and 8 dS m^−1^) and five sett priming doses of SA (0 [hydropriming] [control], 0.5 mM, 1 mM, 1.5 mM and 2 mM) with four replications. Commercial grade NaCl and analytical grade SA (Sigma-Aldrich) was used for preparing solutions as per the treatment requirements. Tap water with 0.5 dS m^−1^ EC was used as control; and 4 dS m^−1^, and 8 dS m^−1^ levels of salinity was obtained by diluting NaCl in tap water at 1.95 g L^−1^ and 4.73 g L^−1^ concentration respectively. Sett priming was done following the procedures described by Islam et al. [23] Surface sterilized setts (10% H_2_O_2_ for 10 min) of the sugarcane variety were soaked into their respective doses of SA for 24 h at room temperature 28 ± 2 °C. Upon completion of priming the primed setts were washed thoroughly with distilled water and air dried under shade on clean dry cloth and sown in the experimental pots. The study was conducted through a germination trial followed by further investigation on early seedling growth. Thirty setts for each treatment combination (5 pots × 6 setts per pot) were placed for the germination trial. The pots were then placed to plastic trays containing saline solution respective to the treatment levels and the pots were also irrigated to field capacity with the same solution. During the whole experimental period the salinity levels in the tray were monitored and maintained using an EC meter (DiST4 portable EC tester, HI98304, Hanna Instruments Inc. USA). Evapotranspiration water loss from every tray was replaced with tap water twice a day. The entire solution of the tray was replaced with fresh one at every two days interval. The setts were monitored daily up to 20 days for germination and from the 21st day onward only one healthy plant per treatment combination was allowed to grow for another 40 days aiming to further investigation in four replications.

### 2.3 Data collection

#### 2.3.1 Sett germination and growth characteristics

Germination parameters on (i) final germination (%) ([*number of germinated setts/total number of setts*] × 100), (ii) germination energy (%) ([*number of germinating setts/number of total setts per treatment after germination for three days*] × 100) [47], (iii) mean germination time (day) ([Σ(*D* × *n*)*/Σn*] where *D* is the number of days from the start of the trial and *n* is the number of setts germinated on day *D*) [48], (iv) seedling length (cm), and (v) seedling vigor index (*seedling length* × *final germination*) [49] was recorded. For seedling length measurement, randomly selected five germinated seedlings per replicate were carefully taken out keeping the roots intact. Excess soil from the roots were separated by washing under running water. Mean value of the five individual lengths measured using a centimeter scale from the tip of the shoot to tip of the furthest root was recorded as seedling length.

Growth parameters data on plant height (cm), total leaf area (cm^2^ plant^−1^), shoot dry matter (g plant^−1^) and root dry matter (g plant^−1^) were collected at 60 days after sett sowing. Plant height (cm) was measured from the soil level to the tip of the furthest leaf using a meter scale. Total leaf area (cm^2^ plant^−1^) was calculated by using the smartphone application Petiole (Petiole Ltd.; https://petioleapp.com/) [50]. Shoot dry matter (g plant^−1^) and root dry matter (g plant^−1^) were recorded by weighing the oven-dried (72 °C) plant shoot and root respectively in an electrical balance after constant weight was achieved. Care was taken while separating the roots from soil to keep the root injury as minimum as possible.

#### 2.3.2 Physiological parameters

Leaf greenness was measured at 60 days after set sowing from the three topmost fully expanded leaves of the plant using a hand held SPAD meter (SPAD-502 plus, Minolta Co. Ltd., Osaka, Japan). Readings were taken randomly from five locations of a leaf and the average value of readings obtained from three leaves was recorded [51].

Relative water content (%) was measured from the middle portion of fully expanded second leaf from the top at 60 days after sett sowing following the procedures of Das et al. [52]. Harvested leaves were immediately stored in airtight zip-lock plastic bags and transported to the laboratory. Leaf disks of 1 cm diameter (excluding midrib) was cut using a disc cutter and thirty disks were weighed in an electrical balance to record their fresh weight (FW). After that the disks were kept immersed in distilled water for 12 h and turgid weight (TW) was measured. Surface moisture of the leaf disks was removed by using blotting paper before recording TW. The leaf disks were then dried in oven (72 °C) till constant weight and their dry weight (DW) was recorded. Finally relative water content (%) was recoded using the formula: Relative water content (%) = $\frac{\left(FW-DW\right)}{\left(TW-DW\right)}\times 100$.

Membrane stability index (%) measurements were also calculated from the middle portion of fully expanded second leaf from the top at 60 days after sett sowing following the procedures of Islam et al. [23]. Leaves were gently rinsed in deionized water and discs of 1 cm diameter (excluding midrib) were prepared using a disk cutter. Thirty leaf disc samples were placed inside airtight vials containing 15 mL deionized water and incubated for 30 min at 40 °C. Electrical conductivity of incubated solutions was measured (EC_1_). Another set of samples same as the first one were boiled at 100 °C for 20 min and electrical conductivity of all samples was measured (EC_2_). Membrane stability index was then calculated using the formula: Membrane stability index (%) = $\left(1-\frac{EC_{1}}{EC_{2}}\right)\times 100$.

#### 2.3.3 Biochemical parameters

Free proline content was measured from the middle portion of fully expanded second leaf from the top following the methods of Bates et al. [53]. Fresh leaf samples were ground into powder with liquid nitrogen, and 0.1 g of the powder was homogenized in 2.0 mL of 10% aqueous sulfosalicylic acid. The aliquot was centrifuged at 10,000 rpm for 10 min and 1 mL of the supernatant was transferred into a test tube containing 1 mL acid ninhydrin solution and 1 mL glacial acetic Acid. The sample was then incubated in boiling water for 1 h, and finally the reaction was terminated in an ice bath. Afterwards, 2 mL toluene was added to the aliquot, mixed vigorously in a vortex mixture at 6000 rpm for 5 min and kept at room temperature for precipitation. The upper layer of the mixture was finally separated for recording spectrophotometer absorbance at 520 nm (SPECORD 50 PLUS UV/Vis Spectrophotometer, Analytik Jena, Germany). Finally, the free proline levels in leaf samples were determined from a standard curve prepared with known concentrations of proline solutions and was expressed as μmol g^−1^ fresh weight.

Total antioxidant activity of the leaf tissue was measured using DPPH (2,2-diphenyl-1-picrylhydrazyl) assay following the method described by Mohammed and Tarpley [54] with slight modification. Thirty leaf discs (20 mm^2^ each) obtained from middle portion of fully expanded second leaf from the top (excluding midrib) were macerated with 10 mL of methanol for 24 h in a sealed test tube at 25 °C under darkness to allow for complete extraction of antioxidants into the solution. Afterwards, 120 μL leaf extract was added to 2880 μL DPPH (0.2 mM) solution and the antioxidant activity of the leaf extract was determined after 4 h by monitoring the reduction in the absorbance of the methanolic solution of DPPH with the extract at 515 nm using a spectrophotometer (SPECORD 50 PLUS UV/Vis Spectrophotometer, Analytik Jena, Germany) against a blank solution of DPPH with pure methanol (zero antioxidant activity). Values were expressed in Trolox equivalents (μmol TE g^−1^ fresh weight) using a Trolox (6-Hydroxyl-2,5,7,8-tetramethylchroman-2-carboxylic acid) and DPPH standard curve.

#### 2.3.4 Mineral ion accumulation parameters

Leaf Na^+^ and K^+^ ion accumulation (mg g^−1^ fresh weight), and Na^+^/K^+^ ratio was estimated as ion content parameters using the LAQUAtwin-Na-11 and LAQUAtwin-K-11 m (HORIBA instruments, CA, USA) respectively following the recommended standards for the instruments. Plant extracts were obtained by homogenizing 0.1 g of fresh leaf samples (taken from the middle portion of fully expanded second leaf from the top) with 10 mL of deionized water. The homogenate was then transferred to airtight tubes and allowed to boil in a hot water bath for 20 min at 120 °C for complete extraction of the ions in the solution. Afterwards, the tubes were allowed to cooldown at room temperature and centrifuged at 10,000 rpm for 10 min. The supernatant was then used for ion content determination (mg L^−1^) and the values were expressed as mg g^−1^ fresh weight. Na ^+^ to K^+^ ratio was calculated using the formula: (Na^+^/K^+^ ratio = Na^+^ ion content/K^+^ ion content).

### 2.4 Statistical analysis

Data obtained were subjected to a two-way analysis of variance (ANOVA) using software program Statistix10 (Analytical Software, Tallahassee, FL, USA). Means of significant treatment effects were separated by conducting post hoc analysis using Tukey’s honest significant difference test. In all analyses, differences were considered significant at *P* ≤ 0.05.

## 3 Results

The two-way ANOVA of the tested parameters considering germination, growth, physiological and biochemical as well as ion contents traits of sugarcane seedlings across the three levels of salinity treatments (S) and five levels of salicylic acid sett priming doses (SA) revealed a significant amount variation across all the treatment combinations (Table 1). Both the individual and interactive effects were highly significant (*P* ≤ 0.01) on all the tested parameters; however, the two-way interaction (S × SA) was found not significant only for germination energy (%). Besides, final germination (%) exhibited significant two-way interactions at *P* ≤ 0.05; whereas the rest of the parameters showed significant two-way interaction at *P* ≤ 0.01 (Table 1).

**Table 1:** Significance level in the two-way ANOVA of the effect of salinity levels and salicylic acid sett priming doses on tested parameters of sugarcane.

Parameters		*P-*value	
	Salinity levels (S)	Salicylic acid doses (SA)	S × SA
**Germination parameters**			
Final germination (%)	0.000	0.000	0.030
Germination energy (%)	0.000	0.000	0.312
Mean germination time (day)	0.000	0.000	0.000
Seedling length (cm)	0.000	0.000	0.000
Seedling vigor index	0.000	0.000	0.008
**Growth parameters**			
Plant height (cm)	0.000	0.000	0.000
Total leaf area (cm^2^ plant^−1^)	0.000	0.000	0.000
Shoot dry matter (g plant^−1^)	0.000	0.000	0.001
Root dry matter (g plant^−1^)	0.000	0.000	0.005
**Physiological and biochemical parameters**			
Leaf greenness (SPAD value)	0.000	0.000	0.000
Relative water content (%)	0.000	0.000	0.000
Membrane stability index (%)	0.000	0.000	0.000
Proline content (μmol g^−1^ fresh weight)	0.000	0.000	0.000
Total antioxidant activity (μmol TE g^−1^ fresh weight)	0.000	0.000	0.000
**Ion contents**			
Na^+^ ion accumulation (mg g^−1^ fresh weight)	0.000	0.000	0.000
K^+^ ion accumulation (mg g^−1^ fresh weight)	0.000	0.000	0.000
Na^+^/K^+^ ratio	0.000	0.000	0.000

*P* ≤ 0.01, *P* ≤ 0.05, and *P* ≥ 0.05 indicates significant at 99% level of confidence, significant at 95% level of confidence and not significant, respectively. SPAD: soil plant analysis development; TE: trolox equivalent.

### 3.1 Responses in sett germination

Proper germination and a good seedling vigor is a prerequisite for better plant growth and development at later stages. In our current study, we recorded a negative trend in performance of the tested germination traits with increasing level of salinity treatments across all the SA priming doses [reduction in final germination (%), germination energy (%), seedling length (cm), and seedling vigor index with an increase in mean germination time (day)]. For example, under 0.5 mM SA treatment level, the final germination (%) decreased 7.7% and 18.0%; seedling vigor index decreased 29.2% and 50.6%; and mean germination time (day) increased 10.1% and 39.7% at 4 dS m^−1^ and 8 dS m^−1^ salinity exposure respectively compared to their control (0.5 dS m^−1^) (Table 2). On the contrary, we recorded a positive trend in performance of the tested germination traits with increasing level of SA sett priming doses across the three salinity levels [increase in final germination (%), germination energy (%), seedling length (cm), and seedling vigor index with a reduction in mean germination time (day)]. For instance, under 8 dS m^−1^ salinity level, the final germination (%) increased 1.3%, 11.5%, 24.8%, and 28.6%; seedling vigor index increased 8.8%, 39.3%, 77.1% and 92.7%; and mean germination time (day) decreased 15.5%, 29.9%, 33.9%, and 35.2% respectively at 0.5 mM, 1 mM, 1.5 mM, and 2 mM SA sett priming doses compared to their control (0 mM) (Table 2). Although salinity exerted negative impacts on overall performance of the tested germination traits, SA priming doses effectively alleviated the negative impacts to a considerable extent. We also found that sett priming with 2 mM SA had the highest alleviation effects across all the salinity treatment levels; however, no significant difference was observed between the effects of 1.5 mM and 2 mM SA priming doses on tested germination traits except for seedling length (cm) particularly under 8 dS m^−1^ salinity level (Table 2). Tested germination traits were adversely impacted by applied salt stress; however, both 1.5 mM and 2 mM SA priming effectively reduced the damages and showed better germination performance as well as seedling vigor index.

**Table 2:** Interactive effects of salinity levels and salicylic acid sett priming doses on germination parameters (final germination, germination energy, mean germination time, seedling length, and seedling vigor index) of sugarcane.

Factor (salinity levels × salicylic acid doses)	Final germination (%)	Germination energy (%)	Mean germination time (day)	Seedling length (cm)	Seedling vigor index
S_0_ × SA_0_	80.42 ± 1.42 abc	47.50 ± 2.85	8.83 ± 0.12 c	13.41 ± 0.09 e	1078.8 ± 15.86 de
S_0_ × SA_0.5_	80.83 ± 1.60 abc	47.92 ± 0.80	8.76 ± 0.18 c	13.87 ± 0.05 d	1120.8 ± 23.79 cde
S_0_ × SA_1_	82.08 ± 2.08 ab	49.58 ± 1.92	8.70 ± 0.14 c	14.33 ± 0.16 c	1176.6 ± 14.73 bcd
S_0_ × SA_1.5_	84.58 ± 0.80 a	50.83 ± 1.60	7.19 ± 0.21 f	17.15 ± 0.20 a	1450.4 ± 15.96 a
S_0_ × SA_2_	85.42 ± 0.83 a	52.92 ± 1.42	6.92 ± 0.20 f	17.40 ± 0.13 a	1485.7 ± 12.99 a
S_4_ × SA_0_	70.42 ± 2.19 cd	37.92 ± 0.79	10.29 ± 0.17 b	10.13 ± 0.11 i	712.6 ± 14.66 h
S_4_ × SA_0.5_	74.58 ± 0.77 a-d	40.00 ± 2.60	9.64 ± 0.14 b	10.63 ± 0.07 h	793.0 ± 13.64 gh
S_4_ × SA_1_	81.25 ± 0.81 abc	45.42 ± 0.77	8.10 ± 0.15 cd	12.83 ± 0.17 f	1042.5 ± 10.68 def
S_4_ × SA_1.5_	83.33 ± 3.33 ab	50.00 ± 1.36	7.32 ± 0.07 def	14.99 ± 0.14 b	1248.4 ± 26.80 bc
S_4_ × SA_2_	85.00 ± 2.15 a	51.67 ± 3.19	7.30 ± 0.13 ef	15.15 ± 0.10 b	1287.7 ± 21.93 b
S_8_ × SA_0_	65.42 ± 0.80 d	32.92 ± 1.42	11.51 ± 0.08 a	7.78 ± 0.08 k	508.8 ± 12.26 i
S_8_ × SA_0.5_	66.25 ± 1.42 d	35.42 ± 0.77	9.73 ± 0.10 b	8.36 ± 0.09 j	553.6 ± 10.19 i
S_8_ × SA_1_	72.92 ± 1.36 bcd	39.58 ± 1.42	8.07 ± 0.32 cde	9.73 ± 0.13 i	708.9 ± 14.40 h
S_8_ × SA_1.5_	81.67 ± 1.69 abc	46.67 ± 2.60	7.61 ± 0.14 def	11.05 ± 0.21 h	901.1 ± 13.65 fg
S_8_ × SA_2_	84.17 ± 2.50 a	50.83 ± 1.70	7.46 ± 0.22 def	11.65 ± 0.06 g	980.5 ± 23.53 ef

Data are means of four replications ± standard error. Means within a column followed by different letters are significantly different based on Tukey’s honest significant difference test at *P* ≤ 0.05. Salinity levels: S_0_ − (0.5 dS m^−1^) [control], S_4_ − (4 dS m^−1^), S_8_ − (8 dS m^−1^); Salicylic acid doses: SA_0_ − (0 mM) [control], SA_0.5_ − (0.5 mM), SA_1_ − (1 mM), SA_1.5_ − (1.5 mM), SA_2_ − (2 mM).

### 3.2 Effects on early seedling growth

Both the salinity treatments and SA priming doses significantly influenced the tested seedling growth parameters at early vegetative growth stage. Regardless of the SA treatment dose, all the growth parameters showed a reduction trend with increasing level of salinity. For instances, under 1.5 mM SA priming level, plant height (cm) showed 1.7% and 16.4% decrease; total leaf area (cm^2^ plant^−1^) showed 6.3% and 14.7% decrease; shoot dry matter (g plant^−1^) showed 12.9% and 19.9% decrease; and root dry matter (g plant^−1^) showed 9.0% and 19.3% decrease due to 4 dS m^−1^ and 8 dS m^−1^ salinity treatment respectively compared to their control (0.5 dS m^−1^) (Table 3). Contrariwise, SA priming doses mitigated the salinity mediated performance decrease in tested growth parameters to a considerable extent while showing an increasing trend in performance regardless of the salinity level. For example, under 8 dS m^−1^ salinity level, the, plant height (cm) increased 30.2%, 50.3%, 61.6%, and 65.7%; total leaf area (cm^2^ plant^−1^) increased 23.1%, 37.2%, 45.8% and 46.6%; shoot dry matter (g plant^−1^) increased 22.8%, 36.3%, 57.6% and 58.2%; and root dry matter (g plant^−1^) increased 30.0%, 42.9%, 67.14%, and 68.6% respectively at 0.5 mM, 1 mM, 1.5 mM, and 2 mM SA sett priming doses compared to their control (0 mM) (Table 3). Salinity treatments had adverse impacts on the tested growth parameters of sugarcane seedlings but SA priming effectively reduced those impacts. The highest applied SA priming dose (2 mM) was found showing the highest alleviation effects across all the salinity treatment levels; however, we did not find any significant difference between 1.5 mM and 2 mM SA priming doses regarding their mitigation effects on the tested growth parameters (Table 3). Although all the tested seedling growth traits showed a dramatic reduction due to salt treatments, both 1.5 mM and 2 mM SA priming significantly reduced the impairments and thereby showed better growth recovery under salinity treatments.

**Table 3:** Interactive effects of salinity levels and salicylic acid sett priming doses on growth parameters (plant height, total leaf area, shoot dry matter, and root dry matter) of sugarcane.

Factor (salinity levels × salicylic acid doses)	Plant height (cm)	Total leaf area (cm^2^ plant^−1^)	Shoot dry matter (g plant^−1^)	Root dry matter (g plant^−1^)
S_0_ × SA_0_	43.50 ± 2.18 d	345.26 ± 4.76 de	5.09 ± 0.04 cde	1.05 ± 0.01 ef
S_0_ × SA_0.5_	44.75 ± 2.41 cd	350.25 ± 3.20 de	5.33 ± 0.10 cd	1.21 ± 0.02 cd
S_0_ × SA_1_	46.58 ± 2.06 b	357.69 ± 3.98 cd	5.82 ± 0.10 ab	1.29 ± 0.01 bc
S_0_ × SA_1.5_	48.93 ± 1.76 a	394.05 ± 4.10 a	6.12 ± 0.13 a	1.45 ± 0.04 a
S_0_ × SA_2_	49.25 ± 2.39 a	400.46 ± 2.60 a	6.21 ± 0.14 a	1.49 ± 0.03 a
S_4_ × SA_0_	37.85 ± 2.14 g	314.58 ± 3.08 g	3.95 ± 0.06 fg	0.82 ± 0.02 h
S_4_ × SA_0.5_	39.88 ± 2.09 f	321.73 ± 3.67 fg	4.69 ± 0.08 e	0.95 ± 0.02 fg
S_4_ × SA_1_	43.68 ± 2.24 d	342.34 ± 1.75 e	4.90 ± 0.08 e	1.05 ± 0.01 e
S_4_ × SA_1.5_	46.08 ± 2.35 bc	369.27 ± 3.36 bc	5.33 ± 0.11 cd	1.32 ± 0.03 b
S_4_ × SA_2_	45.75 ± 2.16 bc	373.05 ± 2.49 b	5.41 ± 0.08 bc	1.35 ± 0.02 b
S_8_ × SA_0_	25.30 ± 2.28 i	230.46 ± 6.96 i	3.11 ± 0.05 h	0.70 ± 0.06 i
S_8_ × SA_0.5_	32.93 ± 2.92 h	283.69 ± 2.86 h	3.82 ± 0.06 g	0.91 ± 0.02 gh
S_8_ × SA_1_	38.03 ± 2.33 g	316.19 ± 1.78 g	4.24 ± 0.09 f	1.00 ± 0.04 efg
S_8_ × SA_1.5_	40.90 ± 2.35 ef	336.11 ± 3.74 ef	4.90 ± 0.12 e	1.17 ± 0.01 d
S_8_ × SA_2_	41.93 ± 1.63 e	337.85 ± 2.55 e	4.92 ± 0.09 de	1.18 ± 0.03 d

Data are means of four replications ± standard error. Means within a column followed by different letters are significantly different based on Tukey’s honest significant difference test at *P* ≤ 0.05. Salinity levels: S_0_ − (0.5 dS m^−1^) [control], S_4_ − (4 dS m^−1^), S_8_ − (8 dS m^−1^); Salicylic acid doses: SA_0_ − (0 mM) [control], SA_0.5_ − (0.5 mM), SA_1_ − (1 mM), SA_1.5_ − (1.5 mM), SA_2_ − (2 mM).

### 3.3 Impacts on physiological and biochemical traits

Physiological parameters like leaf greenness (SPAD value) relative water content (%) and membrane stability index (%) as well as biochemical parameters like proline content (μmol g^−1^ fresh weight) and total antioxidant activity (μmol TE g^−1^ fresh weight) was also highly influenced by the effects of both salinity levels and SA priming doses. In case of physiological traits, both of the leaf greenness (SPAD value), relative water content (%) and membrane stability index (%) showed a significant reduction of values due to increasing salinity levels regardless of the SA priming dose. For example, under 1.5 mM SA priming level, leaf greenness (SPAD value) decreased 1.4% and 5.3%; relative water content (%) decreased 8.3% and 11.9%; and membrane stability index (%) decreased 3.7% and 8.1% at 4 dS m^−1^ and 8 dS m^−1^ salinity treatment respectively compared to their control (0.5 dS m^−1^) (Table 4). On the other hand, we observed an increasing trend in performance of both leaf greenness (SPAD value), relative water content (%) and membrane stability index (%) due to increase in SA priming doses across all the three salinity treatments. For instance, under 8 dS m^−1^ salinity level, leaf greenness (SPAD value) increased 13.6%, 23.7%, 28.8% and 34.4%; relative water content (%) increased 6.5%, 14.6%, 30.7% and 34.8%; and membrane stability index (%) increased 21.5%, 34.8%, 46.9%, and 50.4% respectively at 0.5 mM, 1 mM, 1.5 mM, and 2 mM SA sett priming doses compared to their control (0 mM) (Table 4). Salinity treatments negatively impacted the overall performance of the tested three physiological traits but SA priming doses considerably ameliorated the negative impacts. Our results revealed that, sett priming with 2 mM SA had the highest alleviation effects across all the salinity treatment levels; however, the effects of both 1.5 mM and 2 mM SA priming doses were statistically similar except for membrane stability index (%) (Table 4). Salt treatments reduced leaf greenness, relative water content and membrane stability index substantially which was recovered greatly by both 1.5 mM and 2 mM SA priming.

**Table 4:** Interactive effects of salinity levels and salicylic acid sett priming doses on physiological parameters (leaf greenness, relative water content, membrane stability index, proline content, and total antioxidant activity) of sugarcane.

Factor (salinity levels × salicylic acid doses)	Leaf greenness (SPAD value)	Relative water content (%)	Membrane stability index (%)	Proline content (μmol g^−1^ fresh weight)	Total antioxidant activity (μmol TE g^−1^ fresh weight)
S_0_ × SA_0_	38.43 ± 1.69 a	74.90 ± 1.22 a-d	83.15 ± 1.13 cd	18.63 ± 0.33 g	8.41 ± 0.20 j
S_0_ × SA_0.5_	38.44 ± 1.52 a	77.00 ± 1.01 abc	84.36 ± 1.11 bc	18.45 ± 0.58 g	9.21 ± 0.25 i
S_0_ × SA_1_	39.03 ± 1.55 a	78.01 ± 0.94 ab	85.24 ± 1.16 ab	20.19 ± 0.93 g	9.29 ± 0.26 i
S_0_ × SA_1.5_	39.10 ± 1.29 a	79.67 ± 0.88 a	85.24 ± 0.68 ab	19.38 ± 0.48 g	9.53 ± 0.11 i
S_0_ × SA_2_	39.03 ± 0.97 a	79.16 ± 1.23 a	86.58 ± 1.09 a	20.35 ± 0.83 g	9.60 ± 0.19 i
S_4_ × SA_0_	34.26 ± 1.60 d	62.70 ± 1.11 fg	65.35 ± 1.15 i	35.91 ± 0.34 f	12.07 ± 0.16 h
S_4_ × SA_0.5_	35.85 ± 1.66 bc	64.38 ± 0.97 fg	72.22 ± 1.10 h	48.02 ± 0.84 d	14.24 ± 0.19 g
S_4_ × SA_1_	36.57 ± 1.52 bc	66.69 ± 1.14 ef	74.24 ± 0.60 g	55.08 ± 0.48 c	17.72 ± 0.30 e
S_4_ × SA_1.5_	38.54 ± 1.62 a	73.09 ± 1.15 bcd	82.10 ± 0.99 d	61.65 ± 0.21 b	21.41 ± 0.12 b
S_4_ × SA_2_	38.74 ± 1.89 a	73.65 ± 1.20 bcd	83.22 ± 1.05 cd	63.29 ± 0.63 b	21.91 ± 0.14 b
S_8_ × SA_0_	28.75 ± 1.10 f	53.66 ± 1.05 i	53.34 ± 0.95 j	42.03 ± 0.34 e	16.82 ± 0.17 f
S_8_ × SA_0.5_	32.67 ± 1.65 e	57.16 ± 1.17 hi	64.82 ± 1.16 i	58.22 ± 1.09 c	19.27 ± 0.13 d
S_8_ × SA_1_	35.55 ± 1.56 cd	61.47 ± 2.50 gh	71.91 ± 1.23 h	64.88 ± 0.47 b	20.10 ± 0.36 c
S_8_ × SA_1.5_	37.03 ± 1.65 b	70.15 ± 1.22 de	78.35 ± 1.10 f	70.86 ± 0.43 a	24.70 ± 0.21 a
S_8_ × SA_2_	38.64 ± 1.58 a	72.33 ± 1.45 cd	80.24 ± 1.17 e	71.22 ± 0.91 a	24.90 ± 0.24 a

Data are means of four replications ± standard error. Means within a column followed by different letters are significantly different based on Tukey’s honest significant difference test at *P* ≤ 0.05. Salinity levels: S_0_ − (0.5 dS m^−1^) [control], S_4_ − (4 dS m^−1^), S_8_ − (8 dS m^−1^); Salicylic acid doses: SA_0_ − (0 mM) [control], SA_0.5_ − (0.5 mM), SA_1_ − (1 mM), SA_1.5_ − (1.5 mM), SA_2_ − (2 mM); SPAD: soil plant analysis development; TE: trolox equivalent.

In case of proline content (μmol g^−1^ fresh weight) and total antioxidant activity (μmol TE g^−1^ fresh weight), we found an increasing trend of performance when the three levels of salinity treatments were compared across the five levels of SA priming doses and vice-versa (Table 4). For instances, under 1.5 mM SA priming level, proline content (μmol g^−1^ fresh weight) increased 3.1 and 3.7 times; and total antioxidant activity (μmol TE g^−1^ fresh weight) increased 2.2 and 2.6 times at 4 dS m^−1^ and 8 dS m^−1^ salinity treatment respectively compared to their control (0.5 dS m^−1^) (Table 4). Similarly, under 8 dS m^−1^ salinity level, proline content (μmol g^−1^ fresh weight) increased 38.5%, 54.4%, 68.6% and 69.5%; and total antioxidant activity (μmol TE g^−1^ fresh weight) increased 14.6%, 19.5%, 46.8% and 48.1% respectively at 0.5 mM, 1 mM, 1.5 mM, and 2 mM SA sett priming doses compared to their control (0 mM) (Table 4). The highest amount of boost in both proline content (μmol g^−1^ fresh weight) and total antioxidant activity (μmol TE g^−1^ fresh weight) was recorded under 2 mM SA priming dose regardless of the salinity level; however, the performances of both 1.5 mM and 2 mM SA priming doses were statistically non-significant for the above-mentioned traits (Table 4). Proline content and total antioxidant activity increased due to salt treatment as a tolerance response but their synthesis was significantly boosted by both 1.5 mM and 2 mM SA priming for enhancing salinity mediated damage protection.

### 3.4 Influence on ion contents

**Fig. 1:**
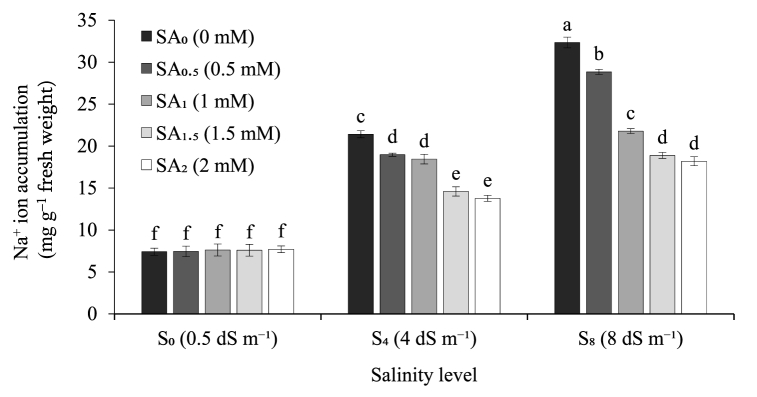
Sodium (Na^+^) ion accumulation by sugarcane seedlings exposed to three salinity levels and five levels of salicylic acid sett priming doses. Data are means of four replications. Vertical bars represent standard error values. Columns with different letters are significantly different based on Tukey’s honest significant difference test at *P* ≤ 0.05. SA: salicylic acid; S: salinity.

**Fig. 2:**
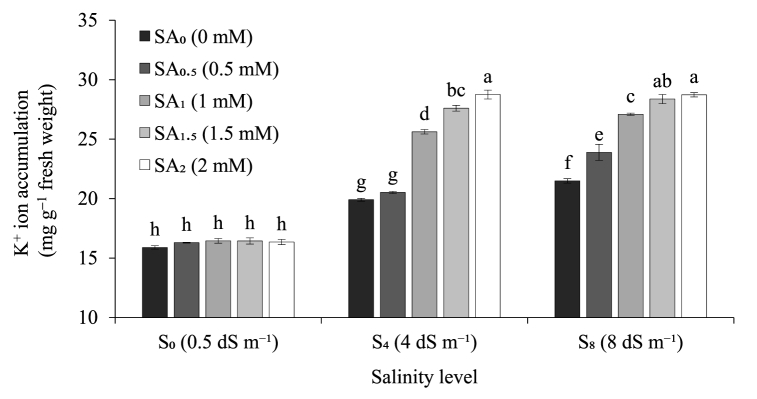
Potassium (K^+^) ion accumulation by sugarcane seedlings exposed to three salinity levels and five levels of salicylic acid sett priming doses. Data are means of four replications. Vertical bars represent standard error values. Columns with different letters are significantly different based on Tukey’s honest significant difference test at *P* ≤ 0.05. SA: salicylic acid; S: salinity.

**Fig. 3:**
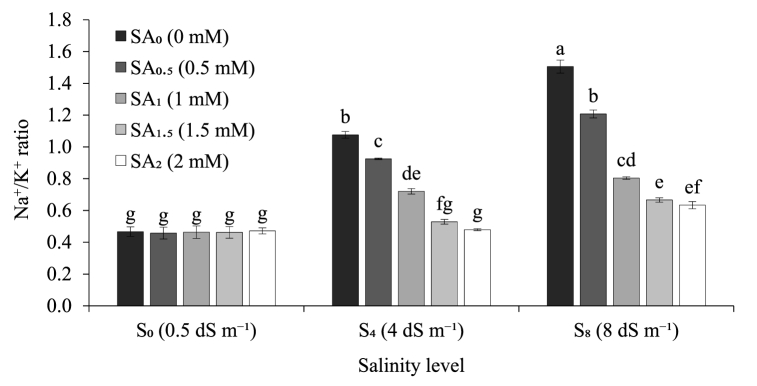
Sodium/potassium (Na^+^/K^+^) ratio of sugarcane seedlings exposed to three salinity levels and five levels of salicylic acid sett priming doses. Data are means of four replications. Vertical bars represent standard error values. Columns with different letters are significantly different based on Tukey’s honest significant difference test at *P* ≤ 0.05. SA: salicylic acid; S: salinity.

Both the salinity levels and SA priming doses greatly impacted on the Na^+^ ion accumulation (mg g^−1^ fresh weight), K^+^ ion accumulation (mg g^−1^ fresh weight), and Na^+^/K^+^ ratio of sugarcane seedlings. In the present study, we observed an increasing rate of Na^+^ ion accumulation (mg g^−1^ fresh weight) with increasing level of salinity treatments regardless of the SA priming dose. For instance, under 1.5 mM SA priming level, Na^+^ ion accumulation (mg g^−1^ fresh weight) increased 1.9 and 2.5 times at 4 dS m^−1^ and 8 dS m^−1^ salinity treatment respectively compared to their control (0.5 dS m^−1^) (Fig. 1). On the other hand, with increasing level of SA priming doses, Na^+^ ion accumulation (mg g^−1^ fresh weight) showed a decreasing trend across all the salinity treatment levels except 0.5 dS m^−1^ (control). For instance, under 8 dS m^−1^ salinity level, Na^+^ ion accumulation (mg g^−1^ fresh weight) decreased 10.8%, 32.6%, 41.6% and 43.8% respectively at 0.5 mM, 1 mM, 1.5 mM, and 2 mM SA priming doses compared to their control (0 mM) (Fig. 1). Similar to the trend observed for Na^+^, the level of K^+^ ion accumulation (mg g^−1^ fresh weight) increased 1.7 and 1.8 times at 4 dS m^−1^ and 8 dS m^−1^ salinity treatment respectively compared to their control (0.5 dS m^−1^) (Fig. 2). However, unlike Na^+^, K^+^ ion accumulation (mg g^−1^ fresh weight) showed an increasing trend with increasing level of SA priming doses across all the salinity treatment levels except 0.5 dS m^−1^ (control). For example, under 8 dS m^−1^ salinity level, K^+^ ion accumulation (mg g^−1^ fresh weight) increased 11.2%, 26.1%, 32.0% and 33.7% respectively at 0.5 mM, 1 mM, 1.5 mM, and 2 mM SA priming doses compared to their control 0 mM SA priming dose. However, under 0.5 dS m^−1^ salinity treatment (control) K^+^ ion accumulation was found similar across all the SA priming doses (Fig. 2). Na^+^/K^+^ ratio of the present study entirely followed the same trend as Na^+^ ion accumulation when the three levels of salinity treatments were compared across the five levels of SA priming doses and vice-versa (Fig. 3). For instance, under 1.5 mM SA priming level, Na^+^/K^+^ ratio increased 15.2% and 45.7% at 4 dS m^−1^ and 8 dS m^−1^ salinity treatment respectively compared to their control (0.5 dS m^−1^) salinity level which was mostly due to comparatively higher amount of Na^+^ ion accumulation than K^+^ ion. On the other hand, with increasing level of SA priming doses, Na^+^/K^+^ ratio showed a decreasing trend as a result of the combined response of reduction in Na^+^ ion accumulation and increase in K^+^ ion accumulation. For example, under 8 dS m^−1^ salinity level, Na^+^/K^+^ ratio decreased 19.7%, 47.0%, 55.6% and 58.3% respectively at 0.5 mM, 1 mM, 1.5 mM, and 2 mM SA priming doses compared to their control (0 mM) (Fig. 3). Among the five doses, 2 mM SA priming performed the best in terms of reducing Na^+^ ion accumulation, increasing K^+^ ion accumulation as well as reducing the Na^+^/K^+^ ratio of the sugarcane seedling; however, we did not record any notable statistical difference between 1.5 mM and 2 mM SA priming doses in terms of in performance for the tested ion accumulation parameters (Fig. 1, Fig. 2, Fig. 3). With the increasing level of salt treatment Na^+^ accumulation increased substantially; however, both 1.5 mM and 2 mM SA priming significantly reduced Na^+^ accumulation, increased K^+^ accumulation and thereby reduced Na^+^/K^+^ ratio.

## 4 Discussion

Sett germination is one of the most crucial phases in sugarcane growth and development. Poor germination results in poor seedling establishment and leads to weak vegetative growth and finally causes a reduction in potential yield. This phase is also among the most susceptible stages to all types of environmental stresses with more possibility of total crop failure than any other crop developmental phases [17]. Soil salinity has already established itself as a highly potent ion toxicity stress that is hampering productivity of a massive portion of world’s croplands concentrating mainly in coastal areas [1,2] through its adverse impacts on plant metabolic functions, bio-membranes, cellular organelles, and important biomolecules via generation of free radicals [5,[10], [11], [12]]. Such effects during initial cropping phase leads to poor germination, seedling establishment and weak seedling vigor, that are bound to exert negative impact on crop performance and productivity. In our present study, we did observe poor final germination, germination energy, seedling length, and seedling vigor index with longer mean germination time due to salt treatments (4 dS m^−1^ and 8 dS m^−1^); however, we also showed that sett priming with SA (particularly 1.5 mM and 2 mM) effectively mitigated such impacts on germination traits to great extents and almost recovered their normal status even under 8 dS m^−1^ salinity level (Table 2). Such performance of SA priming is quite promising for sugarcane cultivation under saline environments as sugarcane is considerably susceptible to salt stress (above 3 dS m^−1^) during its early growth stages [44] and the genotype we used is no different. The result of the current study on germination traits are comparable with earlier studies on SA priming performed in crops like rye, baby corn, pearl millet, wheat and rice [23,[41], [42], [43]]. Salt stress is also well-known to limit plant’s water uptake ability [9]. Presoaking setts wit SA while priming not only facilitate sett hydration but also acts as a signaling molecule during seedling growth that helps combating the ionic stress of salinity through stimulation of metabolic activities [17,[40], [41], [42]]. Our results also indicated the same that sett priming with SA boosted the germination traits as well as tested physiological and biochemical parameters at the early growth stages of sugarcane seedling exposed under differential level of salt stress (Table 2).

Changes in growth attributes is not only an effective measure of crop performance under stressful environments but also important indicators of stress effects [[55], [56], [57]]. Plant height and shoot dry matter accumulation are two of the most important attributes directly related to the yield of sugarcane [58] that demands proper photosynthate production and translocation [59]. However, Plant roots are the first site of salt stress damage which in turn cause improper performance by the aerial parts of the plant due to disruption in the effective water and nutrient uptake [60]. These phenomena were clearly evident in the present study during the early growth of sugarcane seedlings as they displayed significant reduction in both plant height, total leaf area, shoot dry matter and root dry matter accumulation when exposed to differential level of salt stress (Table 3). This trend also explains the underlying effects of both the osmotic and ionic stress components of salinity on the cellular processes, particularly those directly related to division and elongation [21,61]. In our experiment, we recorded reduction as high as 41.8% in plant height, and 33.3% in total leaf area which in turn resulted in 60.1% reduction in shoot dry matter content and 33.3% reduction in root dry matter content due to a salinity exposure of 8 dS m^−1^ (Table 3). Such high damage is bound to have harsh impacts on cane yield in terms of both quantity and quality. However, sett priming with SA effectively and significantly reduced the salt stress damage greatly on the growth parameters. The recovery rate we observed was as high as 65.7% for plant height, 46.6% for total leaf area, 58.2% for shoot dry matter, and 68.6% for root dry matter when applied at 2 mM dose under 8 dS m^−1^ salinity (Table 3). Similar to our findings, Cha-um and Kirdmanee [62] also reported decrease in plant height, shoot weight and leaf area of salt stressed sugarcane plantlets; as well as Badil et al. [44] reported in another study that SA could alleviate the salinity stress effects on total dry matter content of sugarcane seedlings. Both of their findings seem to be in parallel to our findings. Moreover, reports from the similar studies on SA priming for mitigating salt stress impacts on various field crops are also in support of our findings in terms growth recovery [23,38,40,63].

In general, excess ionic stress of salinity is more devastating to plant cells than its osmotic stress component that triggers cell membrane damage, photosynthetic pigment degradation, organelle damage leading to stunted growth, low yield and even complete crop failure in extreme cases for many a crop plants including sugarcane [5,12,62,64]. Furthermore, the oxidative stress via free radicle generation due to salinity holds the potential for intensifying the damages at even greater extents [11]. Since the early growth stages of crops are naturally sensitive towards all kinds of stress including salinity, our observations on salt mediated reduction in growth parameters in such high percentages could possibly be due to salinity induced alterations underlying at cellular level. Contrariwise, the SA priming impacts on salt stress damage mitigation observed on the same growth parameters should also involve protective actions on cellular physiology and biochemical attributes. Thus, in this current study we investigated some of the important physiological and biochemical traits involved in plant salt stress tolerance.

Leaf greenness measured in terms of SPAD value is a non-destructive way of indicating the amount of photosynthetic pigment (chlorophyll) in plant leaves. Our results indicated that, salt stress reduced the greenness of the sugarcane seedlings but the extent of damage was almost fully recovered by both 1.5 mM and 2 mM SA priming doses (Table 4). Salinity mediated chlorophyll decrease was also been previously reported in sugarcane [62] and the protective effects of SA on chlorophyll content against salinity has been reported in many previous studies conducted for field crops like rice, maize and wheat [39,42,43,65,66]; however, for sugarcane such studies are very scarce. Osmotic stress exerted by salinity on sugarcane seedlings reduced the relative water content which was also alleviated considerably by SA priming of sugarcane setts. Responses in membrane stability index was also found similar as relative water content (Table 4). These findings confirm the fact that presoaking setts with SA facilitates both osmotic and ionic stress tolerance via physiological adjustments in the above-mentioned parameters as reported by both Islam et al. [23], Razmi et al. [67] and Sheteiwy et al. [42]. Furthermore, the role of SA in plant growth regulation under salinity via stress signaling and improvement of relative water content, membrane stability, osmolyte biosynthesis, and antioxidant activities has also been previously reported for a number of field crops including sugarcane [3,44,66,[68], [69], [70]].

Maintenance of osmotic balance is a crucial characteristic for normal metabolic activities as well as for normal plant growth and development. Under water stress a well-developed osmoregulation mechanism via synthesis of osmolytes (e.g., proline, glycine betaine, soluble sugars, polyamines) contributes greatly for turgor pressure maintenance [31]. In our current study, we observed a significant boost in proline level due to SA treatment under salt stressed condition (Table 4). According to Misra and Mishra [71], SA could significantly upregulate the activity of proline biosynthesis enzymes (pyrroline-5-carboxylate reductase and γ-glutamyl kinase) and down regulate proline oxidase activity under salinity stress which explains our results. High level of proline accumulation in stressed plants not only helps adjusting cell osmotic balance but also stabilizes bio-membrane integrity, prevents photosystem-II complex disruption, and also detoxifies toxic free radicals [72,73]. These reports also support our findings regarding the recovery of relative water content and membrane stability index as well as also insights about the potential involvement of proline as both an osmolyte and a potential free radicle scavenger under salt stressed condition. Oxidative damages by free radicles in response to excess salt ion accumulation is one of the most devastating results of ionic stress component of salinity. As a natural osmotic stress response plant reduces stomatal aperture to conserve water which ultimately reduce the CO_2_ availability and NADPH consumption of the Calvin Cycle. As a result, ferredoxin of photophosporylative electron transport chain could possibly become oversaturated with electron flow and excess electrons may be transferred from photosystem-I to form free radicals like superoxide (O_2_^•−^), hydroxyl radical (OH•), singlet oxygen (^1^O_2_), hydrogen peroxide (H_2_O_2_) and many more [74,75]. Over production of such free radicals beyond the neutralization capacity of cell can not only disrupt normal metabolism but also potentially damage cellular components like, organelle, membranes, and important biomolecules (lipids, proteins, and nucleic acids) [72,74,76]. Antioxidants of both enzymatic and non-enzymatic nature like superoxide dismutase (SOD), catalase (CAT), peroxidase (POX), polyphenol oxidase (PPO), ascorbate peroxidase (APX), glutathione reductase (GR), ascorbic acid (AsA), tocopherols, flavonoids protect plants from the high oxidative stress damage generated from excess ion accumulation [74,76,77]. For sugarcane, many previous reports suggested that both proline content and antioxidant activities peak high due to salinity exposure [62,64,[78], [79], [80]]. In our investigation we also observed similar responses, such as proline level and total antioxidant activity in salt stressed sugarcane seedlings could naturally reached twice as much than non-stressed seedlings. However, SA priming enhanced their responses even further (3.7 times higher proline content and 2.6 times higher total antioxidant activity) (Table 4). Previous reports suggests that SA could upregulates genes responsible for biosynthesis of heat shock proteins; enzymatic antioxidants like CAT, SOD, POX and glutathione peroxidase (GPX); secondary metabolites and their derivatives like carotenoids, glutathione and tocopherol [81,82]. Very recently both Ahmed et al. [35] and Jannesar et al. [36] reported that SA priming treatment significantly boosted SOD, CAT, POX, PPO, APX, and GR activity in salt stressed plants. These repeated mentions regarding proline and antioxidant activities insights that proline accumulation and antioxidant defense are natural systemic defense response of plants against salt stress; however, SA priming could effectively boost their biosynthesis and activity to induce better salt stress tolerance in sugarcane. Our findings on SA priming mediated boost in proline content and total antioxidant activity is also positively backed by many other earlier reports on various field crops [23,35,46,68,[83], [84], [85], [86]] but we could not find notable mentions specific to sugarcane.

Ionic imbalance of Na^+^ and K^+^ in particular is a common phenomenon in salt stressed plants that remains the key reason for the crop abnormalities [87,88]. A low Na^+^/K^+^ ratio in the cytosol is essential for the normal cellular functions of plants [23]. In our present study, we observed a massive increase in cellular Na^+^ ion accumulation due to exposure of sugarcane seedlings to salinity treatments (Fig. 1). In parallel to that we also recorded that cellular K^+^ ion accumulation also increased substantially along with increasing Na^+^ ion level (Fig. 2) although the increasing rate of was K^+^ ion accumulation remained much lower than Na^+^ ion accumulation for the non-primed seedlings (Fig. 1, Fig. 2). On the contrary, SA primed seedlings not only showed a decrease in cellular Na^+^ ion accumulation but also the K^+^ ion accumulation rate was comparatively higher than Na^+^ ion accumulation which in turn reduced the cellular Na^+^/K^+^ ratio as low as 0.48 (Fig. 1, Fig. 2, Fig. 3). Several previous studies related to SA seed priming mediated salt stress mitigation of different field crops stated similar responses to ours on both Na^+^ and K^+^ ion accumulation [23,41,89,90]. Salicylic acid has previously been reported for modulating Na^+^ ion homeostasis related NHX1, SOS1 and HKT1 and HKT2 expression [91] which could possibly provide reasoning behind the reduction of cellular Na^+^ ion accumulation in SA primed seedlings. In a study conducted by Ahmed et al. [35], showed the involvement of SA in up-regulation of the plasma membrane Na^+^/H^+^ antiporter gene *SOS1* and vacuolar Na^+^/H^+^ exchanger gene *NHX1* under salt stressed condition for better salt ion homeostasis through vacuolar compartmentalization. In another study, SA was found to regulate the expression of isochorismate synthase (*ICS*) gene that helped in inducing systemic salt tolerance in pistachio plant [36]. However, the reason behind the effect of SA priming on boosting cellular K^+^ ion accumulation remained unclear. Several previous studies concluded that, higher cellular K^+^ ion accumulation in salt stressed plants is a natural stress tolerance response functioning via Ca^2+^ and abscisic acid (ABA) mediated regulation of cationic channels present in cellular bio-membranes that shifts their affinity from Na^+^ to K^+^ and thereby maintain low Na^+^/K^+^ ratio [3,82,92]. Moreover, endogenous SA signaling in association with ABA and ethylene was also reported to limit excess Na^+^ influx and promote K^+^ influx in the aerial plant parts via bending the affinity of non-specific cation channels (NSCC) towards K^+^ resulting in better cellular ion homeostasis [31,93]. In our current investigation we also noticed that the level of cellular K^+^ ion accumulation increased significantly under 4 and 8 dS m^−1^ salinity treatment level compared to their control (0.5 dS m^−1^) only up to 1 mM SA priming dose and above that (1.5 mM and 2 mM SA priming doses) the K^+^ ion accumulation for both 4 and 8 dS m^−1^ salinity level is statistically similar. This response is quite unlikely scenario that could possibly have resulted from lower concentration of K^+^ ion compared to Na^+^ ion or unavailability of enough K^+^ ion in the growing media. Thus, adjusting the dose of K^+^ might help boosting the efficacy of SA mediated salt tolerance responses.

In case of all the tested parameters 2 mM SA priming has shown its superiority over other doses although statistically its performance was on par with 1.5 mM SA priming treatment for most of the tested parameters. Apart from that, we also noticed that under 0.5 dS m^−1^ salinity treatment (control), SA priming doses had no to very less significant effect on most of the tested parameters specially, final germination, germination energy, leaf greenness, relative water content, proline content, total antioxidant activity, Na^+^ ion accumulation, K^+^ ion accumulation, and Na^+^/K^+^ ratio. However, at under 4 dS m^−1^ and 8 dS m^−1^ salinity treatment level, significant improvement effects of SA priming doses were clearly evident (Table 2, Table 3, Table 4; Fig. 1, Fig. 2, Fig. 3). Such response insights the specificity of SA functioning in plants under salt stress. Besides, there was considerable significant improvement of seedling length, seedling vigor index, plant height, total leaf area, shoot dry matter, root dry matter and membrane stability index due to different SA priming doses even under 0.5 dS m^−1^ salinity level which not only indicates the beneficial effects of SA on plants but also insights possible involvement of SA with several other plant cellular processes mostly related to plant growth regulation. Further investigation in this area might result in better understanding of SA functioning under osmotic and ionic stress conditions.

## 5 Conclusion

Salt stress resulted in a high cellular Na^+^ ion accumulation of sugarcane seedlings and thereby adversely impacted germination and early growth via impairment of tested physiological and biochemical attributes. However, sett priming with SA effectively mitigated the adverse impacts to a considerable extent and thereby facilitated better germination and early seedling growth under saline environment. SA mediated boost in proline content, total antioxidant activity, and reduction in Na^+^/K^+^ ratio remained as the most important traits safeguarding relative water content, membrane stability and overall plant growth. Among the tested doses, 2 mM SA priming performed best, although both 1.5 mM and 2 mM doses were equally effective from statistical perspective. We are convinced that SA sett priming of sugarcane could mitigated salt stress impairments during the early growth stages; however, crop growing environment especially soil characteristics could potentially influence the alleviation responses. Thus, further investigations focusing on the vegetative and reproductive stage as well as including soil-plant nutrients dynamics is necessary for better understanding. Nevertheless, SA priming could prove as a potential salinity management technique for better crop stand of sugarcane in saline environments.

## Author contribution statement

Tasfiqure Amin Apon: Conceived and designed the experiments; Performed the experiments; Wrote the paper.

Sheikh Faruk Ahmed: Conceived and designed the experiments; Analyzed and interpreted the data; Contributed reagents, materials, analysis tools or data; Wrote the paper.

Zannatul Ferdaous Bony; Md. Rizvi Chowdhury; Jannatul Ferdoushi Asha: Performed the experiments; Analyzed and interpreted the data; Wrote the paper.

Arindam Biswas: Contributed reagents, materials, analysis tools or data; Wrote the paper.

## Data availability

The datasets used and/or analyzed during the current study are available from the first author as well as the corresponding author on reasonable request.

## Funding

This research did not receive any specific grant from funding agencies in the commercial, public, or not-for-profit sectors.

## Ethics approval

Not applicable.

## Consent to participate

Not applicable.

## Consent for publication

Not applicable.

## Declaration of competing interest

The authors declare that they have no known competing financial interests or personal relationships that could have appeared to influence the work reported in this paper
