# Supplementary material for: Sett priming with salicylic acid improves salinity tolerance of sugarcane (Saccharum officinarum L.) during early stages of crop development
Source: Heliyon. 2023 May 6;9(5):e16030. doi: 10.1016/j.heliyon.2023.e16030 (PMC10192769; doi:10.1016/j.heliyon.2023.e16030)
Supplement: Multimedia component 1 [file mmc1.docx]

**Supplementary table 1:** Initial nutrient status of the Ganges-river floodplain soil used in this experiment.

| **Soil properties** | **Observed value** |
| --- | --- |
| Nitrogen (%) | 0.076 |
| Phosphorus (μg g^−1^) | 10.32 |
| Potassium (meq/100g) | 0.46 |
| Calcium (meq/100g) | 8.75 |
| Magnesium (meq/100g) | 2.46 |
| Sulfur (μg g^−1^) | 9.25 |
| Zinc (μg g^−1^) | 1.72 |
| Boron (μg g^−1^) | 0.74 |
| Iron (μg g^−1^) | 14.59 |
| Copper (μg g^−1^) | 0.61 |
| Manganese (μg g^−1^) | 3.42 |
| Molybdenum (μg g^−1^) | 0.28 |
| Organic Carbon (%) | 1.65 |

Soil sampling and analysis was conducted following the procedures described by Estefan et al., (2013).

**Supplementary table 2:** Fertilizers and their doses applied to the experimental pots.

| Nutrient | Application dose  (kg ha^−1^) | Amount per pot  (mg) | Application form |
| --- | --- | --- | --- |
| Nitrogen (N) | 180 | 804 | Urea |
| Phosphorus (P) | 60 | 268 | Triple super phosphate |
| Potassium (K) | 90 | 402 | Muriate of potash |
| Sulfur (S) | 30 | 134 | Gypsum |
| Boron (B) | 5 | 23 | Boric acid |

Calculation of fertilizer rate were performed considering 15 cm soil depth and bulk density of experimental soil was 1.33 g cm^−3^

**Reference**

Estefan, G., Sommer, R., Ryan, J., 2013. Methods of soil, plant, and water analysis. A manual for the West Asia and North Africa region, International Center for Agricultural Research in the Dry Areas (ICARDA) 3, 65-119. Available at: <https://hdl.handle.net/20.500.11766/7512>.
